# Evolutionary history and functional implications of protein domains and their combinations in eukaryotes

**DOI:** 10.1186/gb-2007-8-6-r121

**Published:** 2007-06-25

**Authors:** Masumi Itoh, Jose C Nacher, Kei-ichi Kuma, Susumu Goto, Minoru Kanehisa

**Affiliations:** 1 Bioinformatics Center, Institute for Chemical Research, Kyoto University, Gokasho, Uji, Kyoto 611-0011, Japan

## Abstract

**Background:**

In higher multicellular eukaryotes, complex protein domain combinations contribute to various cellular functions such as regulation of intercellular or intracellular signaling and interactions. To elucidate the characteristics and evolutionary mechanisms that underlie such domain combinations, it is essential to examine the different types of domains and their combinations among different groups of eukaryotes.

**Results:**

We observed a large number of group-specific domain combinations in animals, especially in vertebrates. Examples include animal-specific combinations in tyrosine phosphorylation systems and vertebrate-specific combinations in complement and coagulation cascades. These systems apparently underwent extensive evolution in the ancestors of these groups. In extant animals, especially in vertebrates, animal-specific domains have greater connectivity than do other domains on average, and contribute to the varying number of combinations in each animal subgroup. In other groups, the connectivities of older domains were greater on average. To observe the global behavior of domain combinations during evolution, we traced the changes in domain combinations among animals and fungi in a network analysis. Our results indicate that there is a correlation between the differences in domain combinations among different phylogenetic groups and different global behaviors.

**Conclusion:**

Rapid emergence of animal-specific domains was observed in animals, contributing to specific domain combinations and functional diversification, but no such trends were observed in other clades of eukaryotes. We therefore suggest that the strategy for achieving complex multicellular systems in animals differs from that of other eukaryotes.

## Abstract 2

A rapid emergence of animal-specific domains was observed in animals, contributing to specific domain combinations and functional diversification, but no similar trends were observed in other clades of eukaryotes.

## Background

Protein domains are the basic building blocks that determine the structure and function of proteins, and they may be considered the units of protein evolution. Furthermore, combinations of protein domains provide a broad spectrum for potential protein function [1-4]. Eukaryotic genome sequencing projects have revealed complicated and varied domain architectures [5]. In particular, the number of domains in a protein sequence is greater in higher eukaryotes, which have elaborate multicellular bodies. Sophisticated domain combinations are thought to have contributed to complicated multicellular functional systems, such as cell adhesion, cell communication, and cell differentiation. Here we perform a systematic survey of the eukaryotic genome sequence data currently available to elucidate how domain combinations evolved and how they are related to specific cellular functions in eukaryotes.

It is already known that the number of combinations involving a particular domain is quite varied, and that the distribution of the number of combination partners follows a power law distribution [6-10]. Preference for partner domains in combination varies depending on the domain. Functionally related genes frequently fuse and result in multidomain proteins that have multiple functions [11,12]. In addition, for the three superkingdoms, namely eukaryotes, eubacteria, and archaea, kingdom-specific domains tend to combine within each other [6,7,9], and the domains that emerged later in eukaryotes tend to have a large number of combination partners [8]. These observations are based on comparative analysis of extant eukaryotes or prokaryotes whose genomes have been sequenced. With recent rapid progress in various eukaryotic genome sequencing projects, comparative analysis of the evolutionary relationships among phylogenetic groups of eukaryotes, as opposed to among individual species, has become possible. This allows more detailed examination of the differences among specific domains and their combinations among phylogenetic groups of eukaryotes.

In this work, we focus on the relationship of domain combinations and functional diversification in eukaryotes, with consideration of hierarchical classification based on their phylogenies. We also explore how domains and their combinations are distributed and conserved in each group of eukaryotes. In order to define specific domains and combinations for each phylogenetic group, we modified the method developed by Mirkin and coworkers [13], which estimates ortholog contents of ancestral species based on the most parsimonious method. The most parsimonious method is a commonly used approach to estimating ancestral ortholog content [14-18].

Our analysis uncovers differences in specific domains and their combinations among different phylogenetic groups of eukaryotes. We observe a large number of animal-specific and vertebrate-specific domain combinations. However, those domains having a large number of combination partners are different in animals and vertebrates, and their functions are strongly linked to their characteristic functions that evolved in the common ancestors of animals and vertebrates. Examples include animal-specific combinations in tyrosine phosphorylation systems and vertebrate-specific combinations in complement and coagulation cascades. In animals, especially in vertebrates, the average connectivity of animal-specific domains is markedly high. In contrast, the older domains tend to have greater average connectivity in other groups of eukaryotes. These observations suggest that the properties of domains are nonuniform in terms of generating domain combinations.

Our findings also made it possible to reconstruct an evolutionary history of the domain combinations in each clade of eukaryotes and to observe changes of combinations based on a global network analysis. The global features of the reconstructed evolution of the network are consistent with the observed differences in properties of group-specific domains. Therefore, our analysis enables us to link local differences among group-specific domains with the global features of domain combination changes during evolution. From these observations, it is suggested that the strategy for achieving complex multicellular systems might be different, even among eukaryotes, in terms of the preference for generation of domain combinations.

## Results

### Assignment of domains and their combinations

**Figure 1:**
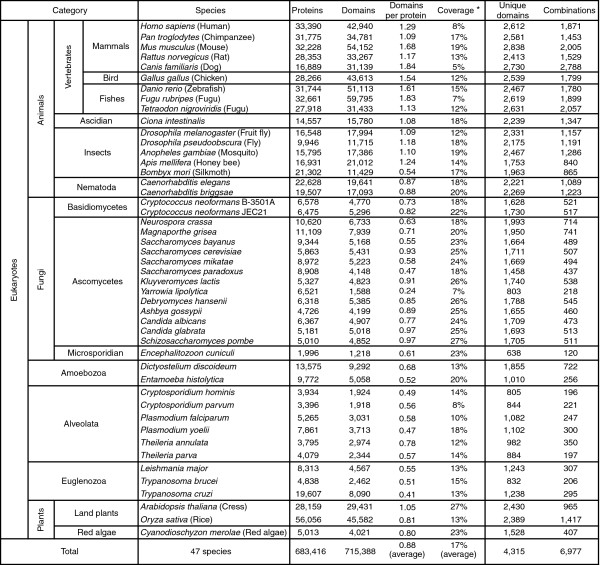
Hierarchical classification and the numbers of domains and domain combinations found in each species. Hierarchical classification of eukaryote groups and results for assignment of Pfam domains are summarized. Additional information is provided in Additional data file 7 (Supplementary Table 1). *Coverage = all residues covered by Pfam domains/all residues.

We used the domains defined in the Pfam database [19]. Of 7,459 domains stored in its Pfam-A section (version 14.0), 4,315 were assigned to the protein sets of 47 eukaryotes, including vertebrates, insects, worms, fungi, plants, and protists. Figure 1 summarizes the hierarchical classification of these eukaryotes based on their phylogenetic relationships and the number of domains found in them (Additional data file 7 [Supplementary Table 1]). In almost all eukaryotic species, Pfam domains covered on average about 10% to 30% of sequence length in each protein set. The coverage did not greatly differ among phylogenetic groups, except for fungi, which had slightly greater coverage. The average number of domains in each protein in higher animals was generally greater than those of other species.

**Figure 2:**
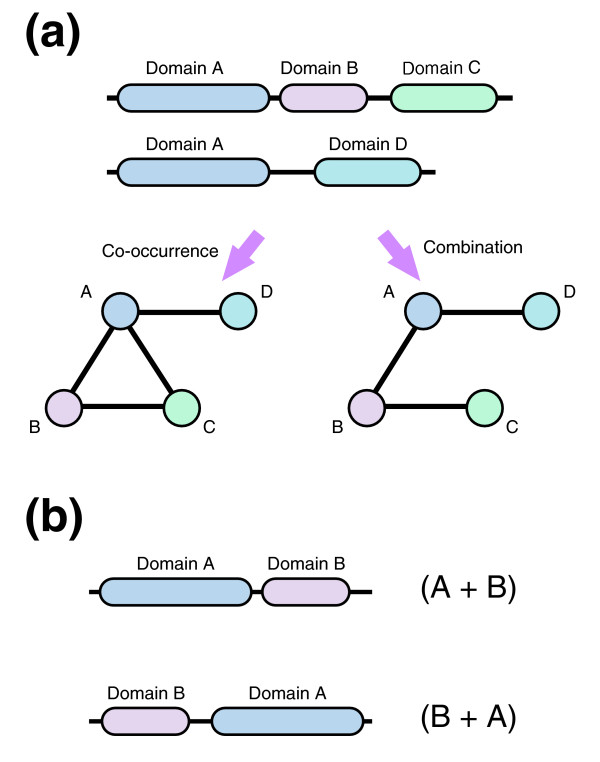
Domain combination. **(a) **Domain architectures in a protein set can be represented as a network. A domain corresponds to a node, and edges refer to the co-occurrence or combination of a domain in the protein set under consideration. In a domain co-occurrence network, two domains are connected by an edge if they co-occurred in the same protein sequence. Here, we considered a domain combination network in which two domains must be located consecutively. Domain B is located between domains A and C, and so nodes A and C are not connected. **(b) **Combinations (A + B) and (B + A) are distinguished in this work.

Domain combinations can be defined in several ways, such as by co-occurrence in a protein sequence. Here, in order to distinguish domain architectures possibly generated by individual evolutionary events, we defined a combination as two consecutively located domains (Figure 2a). We also distinguished between combinations when the order of two domains on a protein was inverted (Figure 2b). In total, 6,977 unique combinations were found in the 47 eukaryote protein sets (Figure 1). The number of domain combinations found in multicellular animals was large (>800), as well as in the multicellular fungi (*Neurospora crassa *and *Magnaporthe grisea*), land plants (*Arabidopsis thaliana *and *Oryza sativa*), and *Dictyostelium discoideum *(about 700 to 1,500). It should be noted that species with a large number of proteins do not always have a large number of domain combinations; for instance, *Entamoeba histolytica *and *Trypanosoma cruzi *have large numbers of proteins and few combinations.

### Estimation of group-specific domains and combinations

**Figure 3:**
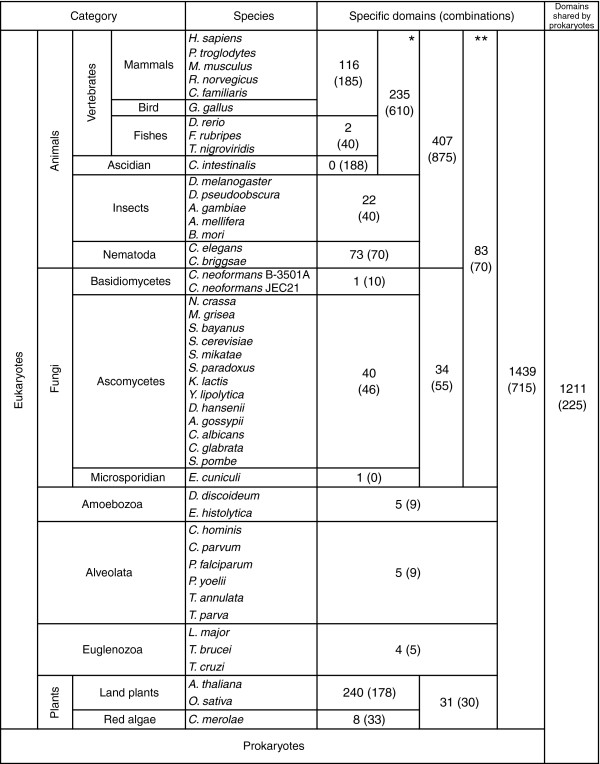
The numbers of group-specific domains and combinations. Summarized are the specific domains and combinations for respective groups of eukaryotes. We consider two additional phylogenetic groups: *Deuterostomes and **Opisthokonta. Some eukaryote genome sequences are still in draft and the number of proteins was smaller than estimated (such as *C. familiaris*). However, our method to define group specificity using the multifurcated phylogenetic tree can reduce effects of incompleteness of genome sequences. Additional information is provided in Additional data file 7 (Supplementary Table 2).

We first identified eukaryote-specific domains in the set of 4,315 domains found in 47 eukaryotes, among which 2,065 domains were also found in prokaryotes. Even if a domain is found in both prokaryotes and eukaryotes, it may still be considered a eukaryote-specific domain in the case of horizontal transfer from eukaryotes to prokaryotes. In order to discriminate those domains that presumably existed in the commonote, the common ancestor of eukaryotes and prokaryotes, we reconstructed the most parsimonious scenario of gains and losses of domains during prokaryotic evolution using the method proposed by Mirkin and coworkers [13]. As a result, 1,211 domains were assigned to the commonote (shown as shared by prokaryotes in Figure 3), and 3,104 domains were considered to be eukaryote specific.

We next identified group-specific domains for each group of eukaryotes, where 47 eukaryotes were divided into 14 groups. We classified the groups hierarchically, based on their phylogenetic relationships (for further details, see Additional data file 1). We considered two additional groups, namely deuterostomes (vertebrates plus ascidian) and opisthokonta (animals plus fungi), in the hierarchical classification. Because horizontal gene transfer among eukaryotes can be disregarded [14,15,20], we assigned the domain to the ancestral group when derived groups and species possess the domain. Among 3,104 domains in eukaryotes, 1,439 domains were shared in all eukaryotes, but the rest were group specific (Figure 3). We observed greater numbers of group-specific domains in higher multicellular eukaryotes: animals, deuterostomes, and land plants.

We then examined group-specific domain combinations. In contrast to the case of group-specific domains, a group-specific combination cannot be defined by simply tracing the last common ancestor because identical combinations can arise independently in different groups. We again used the method proposed by Mirkin and coworkers [13] to reconstruct the most parsimonious scenario and estimated that only 128 combinations were generated in multiple groups. In Figure 3, we show the number of group-specific combinations in the major eukaryote groups (also see Additional data file 7 [Supplementary Table 2]). In animals and deuterostomes, the numbers of group-specific domain combinations were large, at 875 and 610, respectively, in addition to the large numbers of group-specific domains themselves. On the other hand, the number of combinations specific to land plants was small compared with the number of specific domains.

### Characterization of animal- and deuterostome-specific domain combinations

Here we focus on the domains forming these animal-specific or deuterostome-specific combinations. The 875 animal-specific combinations consist of 558 domains, and the 610 deuterostome-specific combinations consist of 478 domains. Among them, 72 domains in animal-specific combinations and 50 domains in deuterostome-specific combinations have more than five partner domains, which we call hub domains. Although 36 domains were commonly found in both groups, the hub domains tend to have preferentially large numbers of combination partners in each group. For example, the protein kinase domain (Pfam ID: Pkinase) was found in 37 animal-specific combinations but only in eight deuterostome-specific combinations. In Tables 1 and 2 we list the hub domains that were preferentially found in animal-specific or deuterostome-specific combinations, respectively.

**Table 1:** The Pfam domains having many combination partners in animal-specific combinations

Pfam ID	Number of partners	Group specificity	Definition
Pkinase	37	Com	Protein kinase domain
SH2	19	Euk	SH2 domain
Laminin_EGF	18	Euk	Laminin EGF-like (domains III and V)
C1_1	17	Euk	Phorbol esters/diacylglycerol binding domain (C1 domain)
RA	12	Euk	Ras association (RalGDS/AF-6) domain
Spectrin	11	Euk	Spectrin repeat
PSI	11	Euk	Plexin repeat
C1_3	10	Euk	C1-like domain
PID	09	Ani	Phosphotyrosine interaction domain (PTB/PID)
Homeobox	09	Euk	Homeobox domain
zf-B_box	08	Euk	B-box zinc finger
LRRNT	08	Ani	Leucine rich repeat amino-terminal domain
zf-MYND	07	Euk	MYND finger
RasGEF	07	Euk	RasGEF domain
DEAD	07	Com	DEAD/DEAH box helicase
cNMP_binding	06	Com	Cyclic nucleotide-binding domain
Y_phosphatase	06	Euk	Protein-tyrosine phosphatase
WAP	06	Ani	WAP-type (whey acidic protein) 'four-disulfide core'
UBA	06	Com	UBA/TS-N domain
ResIII	06	Com	Type III restriction enzyme, res subunit
PWWP	06	Euk	PWWP domain
MIB_HERC2	06	Euk	Mib_herc2
LRRCT	06	Ani	Leucine rich repeat carboxyl-terminal domain
LIM	06	Euk	LIM domain
KH_1	06	Com	KH domain
HECT	06	Euk	HECT-domain (ubiquitin-transferase)
DUF1136	06	Ani	Repeat of unknown function (DUF1136)
Band_41	06	Euk	FERM domain (Band 4.1 family)

Shown are hub domains preferentially found in animal-specific combinations. We defined hub domains that are preferentially found in animal-specific combinations as those found in animal-specific combinations more than twice as frequently as in deuterostome-specific combinations. Regarding the group specificity of the domains, the terms 'Euk', 'Ani', and 'Deu' refer to eukaryote, animal, and deuterostome, respectively. 'Com' indicates that the domain is shared by prokaryotes and eukaryotes.

**Table 2:** The Pfam domains having many combination partners in deuterostome-specific combinations

Pfam ID	Number of partners	Group specificity	Definition
VWA	14	Com	von Willebrand factor type A domain
WD40	13	Euk	WD domain, G-beta repeat
MAM	12	Euk	MAM domain
SAM_2	11	Euk	SAM domain (sterile alpha motif)
Lectin_C	11	Euk	Lectin C-type domain
Kunitz_BPTI	11	Ani	Kunitz/Bovine pancreatic trypsin inhibitor domain
Collagen	11	Euk	Collagen triple helix repeat (20 copies)
WW	10	Euk	WW domain
TIL	10	Ani	Trypsin Inhibitor like cysteine rich domain
IQ	10	Euk	IQ calmodulin-binding motif
Trypsin	09	Com	Trypsin
GPS	08	Ani	Latrophilin/CL-1-like GPS domain
GCC2_GCC3	08	Euk	GCC2 and GCC3
Death	08	Ani	Death domain
CH	08	Euk	Calponin homology (CH) domain
zf-RanBP	07	Euk	Zn-finger in Ran binding protein and others
fn2	07	Deu	Fibronectin type II domain
Xlink	07	Deu	Extracellular link domain
F5_F8_type_C	07	Euk	F5/8 type C domain
zf-CCCH	06	Euk	Zinc finger C-x8-C-x5-C-x3-H type (and similar)
Kringle	06	Euk	Kringle domain
Kazal_2	06	Euk	Kazal-type serine protease inhibitor domain
Kazal_1	06	Euk	Kazal-type serine protease inhibitor domain

Shown are hub domains preferentially found in deuterostome-specific combinations. We defined hub domains that are preferentially found in deuterostome-specific combinations as those found in deuterostome-specific combinations more than twice as frequently as in animal-specific combinations. Regarding the group specificity of the domains, the terms 'Euk', 'Ani', and 'Deu' refer to eukaryote, animal, and deuterostome, respectively. 'Com' indicates that the domain is shared by prokaryotes and eukaryotes.

These hub domains in group-specific combinations are presumably involved in different functions that have evolved in the common ancestors of respective groups. In animal-specific combinations, the protein kinase domain (Pkinase) was found to have the greatest number of partners. Other hub domains in animal-specific combinations include the SH2 domain, the protein-tyrosine phosphatase domain (Y_phosphatase), and the phosphotyrosine interaction domain (PID), which are all related to tyrosine phosphorylation signaling (Table 1) [21-24].

On the other hand, domains involved in the complement and blood coagulation cascade were frequently found in deuterostome-specific combinations (Table 2). In the complement and blood coagulation cascade, the trypsin-like serine protease domain plays an important role, and the cascade is distributed among species in deuterostomes. We observed the trypsin-like serine protease domain (Trypsin) and its inhibitors (TIL, Kazal_1, Kazal_2, and Kunitz_BPTI) as hub domains in deuterostome-specific combinations. Furthermore, other domains involved in the cascade, such as von Willebrand factor type A domain (VWA), Lectin (lectin_C), F5/8 type C domain (F5_F8_type_C), and kringle domain, were also hub domains in deuterostome-specific combinations.

### Group-specificity and connectivity of domains

**Figure 4:**
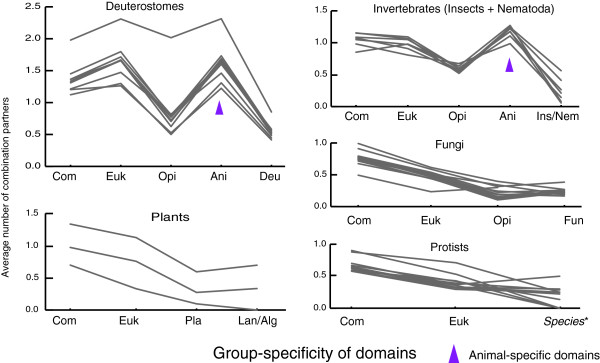
The average number of combination partners of group-specific domains. This figure illustrates the difference in the number of combination partners among each group-specific domain in extant species. Each line shows average number of combination partners of group-specific domains in extant species in deuterostomes, invertebrates, fungi, plants, and protists. Euk, Ani, Opi, Deu, Pla, Fun, Lan, Alg, Ins, and Nem refer to eukaryote, animal, opisthokonta, deuterostome, plant, fungus, land plant, alga, insect, and nematode specific domains, respectively. Com indicates the domain shared by eukaryotes and prokaryotes. These are ordered along with the hierarchy of species, which implies the age of domains. Domains in Deu, Fun, Lan, Ins, and Nem also include domains specific to respective subgroups of them because these numbers are very small. Species* in the graph of Protists refers to each group of protists such as alveolata and euglenozoa. The outlier in Deuterostomes (*C. familiaris*) reflects the incompleteness of its its genome sequence, and the difference among distributions for three plants reflect their distant evolutionary relationship. The hierarchical classification of groups and the numbers of their specific domains are shown in Figure 3, and all information for respective species and group-specific domains is provided in Additional data files 2 to 6.

Figure 3 shows the numbers of group-specific combinations, including 875 animal-specific and 610 deuterostome-specific combinations, in the hierarchical classification of phylogenetic groups. To inspect contributing factors for generating large numbers of domain combinations during the course of evolution, we examined the number of combination partners of group-specific domains plotted against the hierarchy of phylogenetic groups (Figure 4). The average number of combination partners is plotted for individual species in the groups of deuterostomes, plants, invertebrates, fungi, and protists. First, as shown in the figure, different species within each group exhibited similar variations. Second, the nonanimal groups (plants, fungi, and protists) exhibited decreasing partners along the hierarchy, indicating that the average number of combination partners of older domains is generally higher than that of new domains. Third, the animal groups (deuterostomes and invertebrates) exhibited characteristic variation patterns. The average number of combination partners of animal-specific domains is much higher in animals, especially in deuterostomes. On the other hand, the number of partners of deuterostome-specific domains is small, despite the large number of deuterostome-specific combinations. These observations indicate that the animal-specific domains (not the deuterostome-specific domains) largely contributed to the emergence of new group-specific combinations in deuterostomes or invertebrates.

### Global features of domain combination networks

**Figure 5:**
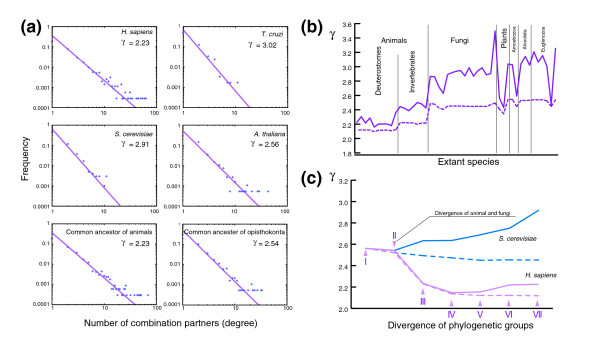
Changes of domain combination networks during evolution. **(a) **Log-log plot of the degree distribution i.n the domain combination networks of *H. sapiens*, *T. cruzi*, *S. cerevisiae*, *A. thaliana*, and estimated ancestral species. Dots represent empirical data, and lines and values of γ were obtained by least squares fitting of the cumulative distribution. **(b) **Difference between domain combination networks of extant species and their union networks. The bold line indicates the values of γ for domain combination networks of extant species, and the dashed line indicates the values for union networks. **(c) **Changes of domain combination networks and union networks in lineages of *S. cerevisiae *and *H. sapiens *during evolution. Bold and dashed lines indicate γ of domain combination networks and union networks, respectively, for estimated ancestors and extant species. It should be noted that the horizontal axis does not indicate the actual time in evolution but the divergence points of each lineage. I to VII indicate the last common ancestors at each divergence point in the *H. sapiens *lineage and suggest divergence times as follows: I, opisthokonta-plant-protist (1,230 to 1,250 million years ago); II, animal-fungi (965 to 1,050 million years ago); III, deuterostome-protostome (656 to 750 million years ago); IV, mammal-fish (350 to 450 million years ago); V, primate-rodent (80 to 90 million years ago); VI, human-chimpanzee (6 to 7 million years ago); VII, extant human [33-36]. Unexpectedly, the periods between divergence points turned out more or less the same (200 to 300 million years), except for the period between VI and VII.

The mechanisms for generating domain combinations was subjected to global network analysis. The decreasing pattern for the nonanimal groups shown in Figure 4 is consistent with preferential attachment to more connected nodes, but the variation pattern for the animal groups may reflect a more complex mechanism. In a domain combination network, an individual domain is represented as a node, and their combination is represented as an edge. Many biologic networks exhibit scale-free properties [25-27], and the domain combination network is no exception [6-10]. The number of domains that combine with a particular domain follows a power law distribution - *p*(*k*) ∝ *k*^-γ ^- where *k *is the number of combination partners (the degree of a node). The degree distributions of combination networks of all domains in *Homo sapiens*, *Saccharomyces cerevisiae*, *A. thaliana*, and *T. cruzi *are shown in Figure 5a, and the values of γ for all species are shown as a bold line in Figure 5b (also see Additional data file 7 [Supplementary Table 2]). As previously reported [8,10], the γ values varied among major groups of eukaryotes. From possible domain combinations of ancestral species estimated using the method of Mirkin and coworkers [13], the degree distributions can be obtained for ancestral species. Figure 5a shows such distributions for the common ancestor of animals and that of opisthokonta (animals plus fungi).

Using this procedure we traced the changes of the γ value along the phylogenetic hierarchy for animals and fungi (Figure 5c; also see Additional data file 7 [Supplementary Table 2]). In the lineage of *H. sapiens *the γ value rapidly decreased after the divergence of animal and fungi, whereas in the lineage of *S. cerevisiae *the γ value gradually increased. In order to examine this difference, we defined the union domain combination network in each lineage of *H. sapiens *and *S. cerevisiae*. All nodes and all edges were accumulated in the union network along the phylogenetic hierarchy without considering the loss of domains or combinations. The γ values for the union networks are shown in dashed lines in Figure 5c, indicating a much greater decrease for the lineage of *S. cerevisiae*. Similar analyses were performed for all other lineages and the result is indicated by the dashed line in Figure 5b. Fungi and protists apparently exhibit a large decrease in γ value in the union network, probably reflecting a large number of gene losses.

## Discussion

### Specific domain combinations in animals and deuterostomes

Using the 47 eukaryotic genomes now available, we were able to analyze protein domains and their combinations that are specific to different phylogenetic groups of eukaryotes. The number of domains per protein increased in higher multicellular species, especially in animals (Figure 1). We also observed large numbers of animal-specific or deuterostome-specific domain combinations (Figure 3). These observations indicate a rapid increase in complexity in domain architecture, which is termed 'domain accretion' [5].

Analyzing the hub domains in these group-specific combinations, we found that domain architectures became more complex within the systems that rapidly evolved in the common ancestors of animals and of deuterostomes (Tables 1 and 2). In animals, protein tyrosine phosphorylation mediated by protein tyrosine kinase plays a crucial role in the processing of signals from the environment and in the regulation of various cellular functions that were developed in early animals. In contrast, in the deuterostome-specific combinations, we found many hub domains involved in the complement and blood coagulation cascade, which is commonly known as a deuterostome-specific innate immune system involving serine protease [28,29]. Note that invertebrates, such as arthropods, also have an independently evolved innate immune system that involves serine protease, but its molecular mechanism is different from that of deuterostomes [30,31].

As shown in Figure 4, animal-specific domains largely contributed to the increase in these animal-specific or deuterostome-specific combinations. In previous reports it was suggested that rearrangement of existing domains in new combinations facilitated evolution of complex systems in multicellular organisms [32]. However, our results indicate that the emergence of highly connected animal-specific domains was essential for the evolution of animals. In contrast, there are no highly connected domains in other multicellular species such as land plants and multicellular fungi, although they actually have a large number of domain combinations. Therefore, in nonanimal multicellular eukaryotes, an increase in complexity of domain architecture did not depend on new group-specific domains. However, the number of sequenced plant and multicellular fungi genomes is still very small, and further analysis taking phylogenetic relationships into consideration will refine our observations.

### Alternative definitions of domains and combinations

Pfam domains are defined based on biologic knowledge. Thus, the criteria for defining sequence families differ from one domain to another depending on the granularity of knowledge regarding the domain. For example, some domains that were grouped together in the past have been categorized separately in newer versions of Pfam because of increased knowledge regarding that domain. Because group specificity of the Pfam domains is affected by these subfamily classifications, this granularity may have affected our results. Therefore, we examined the consistency of our results by using different definitions of domains in which we hierarchically classified eukaryote-specific Pfam domains into more granular subfamilies (see Materials and methods, below).

Table 3 shows the number of each group-specific subfamily of eukaryote-specific domains as well as combination partners that are unique to each group-specific subfamily. As shown here, the increase in unique combination partners of eukaryote-specific domains also occurred after the divergence of animal-specific subfamilies. In the other direction, we also examined lax definitions of domains by merging Pfam domains according to evolutionary relationships based on Pfam Clans [19] and all trends were conserved (data not shown). From these observations, we claim that our results do not depend on the granularity of the domains.

**Table 3:** The number of subfamily divergences of eukaryote-specific domains

Groups	Subfamily duplications	Combination partners	Duplicated domains
Opisthokonta	848	219	164
Animals	2,735	713	363
Deuterostomes	3,902	487	323
Mammals + bird	3,394	166	226
Primates	1,226	010	081

Each row corresponds to a particular group; shown are the number of subfamilies duplicated and the number of unique combination partners for subfamilies duplicated in the group. The 'Duplicated domains' column indicates the number of domains that were duplicated in the group.

For completeness, we further analyzed the affect of the definition of the domain combination networks on our results. In related work, domain combination networks were simply defined as the co-occurrence of two domains in a protein sequence without considering domain order. Using this definition, all trends in our results were conserved (data not shown).

### Comparison with previous findings on the connectivity of domains

Wuchty [8] indicated that the connectivity of domains did not correlate with their age and that domains with high connectivity emerged late in eukaryote evolution. These observations were based only on results from a comparison of prokaryotes, *S. cerevisiae*, *Caenorhabditis elegans*, and *Drosophila melanogaster*. Therefore, the results indicating high connectivity in late eukaryotes could not be generally claimed; high connectivity was actually found mostly in animals, and not necessarily in fungi and plants. In animals, we also found that the animal-specific domains have very high connectivity, which correlated well with their work. However, when considering group-specific domains in nonanimal groups, we observed a correlation between connectivity and age, in which the oldest domains inherited from the commonote had the greatest connectivity among nonanimal eukaryotes (Figure 4). Note that we computed connectivity based on the average domain connectivity for each age. That is, although in principle older domains had more combination partners, domain combinations differed depending on domain or clade identity, and as a result we could obtain these correlations between connectivity and age.

### Linking molecular analysis and network analysis

By tracing and comparing the changes of domain combination networks together with the phylogenetic relationships between eukaryotes, we observed differences in the evolution of the combination networks in *H. sapiens *and *S. cerevisiae *(Figure 5c). In the *H. sapiens *lineage, the γ value decreased after the divergence of animals from fungi. Evolutionary analysis using molecular clock and fossil data suggests that the period between animal-fungi divergence and deuterostome-invertebrate (insects plus nematoda) divergence was about 300 million years, and that the lengths of the periods differed little from each other [33-36] (see the legend to Figure 5c). It is therefore suggested that the decrease of the γ value occurred rapidly. Such growth concurrent with the decrease of γ is called accelerated growth, which is a general and widespread feature of growing networks [37,38]. Accelerated network growth during animal evolution is due to the high connectivity of animal-specific domains.

In the *S. cerevisiae *lineage, the γ value of the domain combination network increased, whereas that of the union network decreased. These observations suggest that there were more complicated domain networks in the ancestral species of fungi, and gene loss strongly affected network evolution in the *S. cerevisiae *lineage. In our dataset, most fungi are unicellular yeasts, and it is suggested that the size of the yeast genomes diminished by gene loss events during evolution [39]. Similarly, the difference between the γ value of domain networks and that of union networks in protists was large, which can also be explained by gene loss events. Many of the protists are parasitic, and it is suggested that they have come to depend on their hosts, in the process losing a number of genes [40-43].

To describe the scale-free behavior and evolutionary mechanisms of various biologic networks, evolutionary models have often been studied [44-48]. The simplest of these models is the preferential attachment model [49], in which new nodes link to an existing node with a probability proportional to its degree. In this model, older nodes have greater connectivity, and the degree distribution is conserved during network growth. However, our results show that the degree distributions were not conserved during evolution because of the accelerated growth in animals and the diminished genome in fungi. Moreover, the connectivity of animal-specific domains was very high (although, in nonanimal groups, average connectivity could be correlated with the age of specific domains). This apparent disagreement is supported by findings reported by Przytycka and coworkers [50,51]; they found the topologic structure of the observed co-occurrence network of real biological data was to be different from synthetically generated random scale-free networks constructed according to the preferential attachment model.

Our findings indicate that the changes in domain combinations differed between periods of evolution as well as among phylogenetic groups, implying that the evolutionary driving force for domain combination generation changed during eukaryotic evolution. Therefore we claim that simple comparison of extant species using a uniform model is insufficient in this case. Consequently, individual species lineages, periods of evolution, and differences in domain propensity for generating combinations must all be taken into consideration.

## Conclusion

Comparison of group specificities of domains and their combinations in different phylogenetic groups of eukaryotes revealed nonuniform properties that could be strongly correlated with the characteristics and evolution of the respective groups. In plants, fungi, and protists, more ancestral domains tend to be reused as hub domains, but the domains that emerged early in animals tend to have large numbers of combination partners. These domain combinations apparently contributed to the functional diversification of animals, including the tyrosine phosphorylation signaling and the coagulation cascades. The distinction of animal and nonanimal groups also helps reconcile two previously reported conflicting views on preferential attachment in the evolution model for the domain combination network.

## Materials and methods

### Proteins, domains, and phylogenetic relationship

We used the proteomes of 47 eukaryotes and 223 prokaryotes obtained from the genome and draft genome sequences stored in the Kyoto Encyclopedia of Genes and Genomes (KEGG) GENES and DGENES databases [52] and the Ensembl database [53] (Figure 1 for eukaryotes). The domains of the protein sequences were assigned based on the Pfam database using the HMMER package [54,55] with threshold *E *value below 10^-3^. When two or more domains overlapped (>50% of the shortest domain length) on a protein sequence, we selected the domain with the most significant *E *value. We used precomputed HMMER results stored in KEGG Sequence Similarity Database (SSDB) with Pfam ver. 14 for protein sequences in KEGG GENES, and we computed the HMMER assignments for proteins obtained from KEGG DGENES and Ensembl with the same Pfam version as stored in KEGG SSDB.

To define specific domains and combinations for each clade of eukaryotes that are hierarchically classified (Figure 1), we consider the most parsimonious scenario of gains and losses of domains and their combinations by considering phylogenetic trees for eukaryotes and prokaryotes. Because of the uncertainty of some phylogenetic relationships and the low coverage rate of the draft genomes, we used multifurcated trees. We inferred a multifurcated consensus tree among 47 eukaryotes based on the recent view of eukaryotic evolution [56,57] as shown in Additional data file 1. On the other hand, there was no clear consensus regarding the relationships among prokaryotes. Therefore, the phylogenetic tree for prokaryotes was inferred from 16S ribosomal RNA sequences and was arranged as a multifurcated tree.

### The most parsimonious scenario with multifurcated trees

Although it is commonly believed that a new gene emerges only once in a single lineage during evolution, genes can also be gained through horizontal gene transfer [58]. Mirkin and coworkers [13] developed an algorithm to estimate the most parsimonious scenario by taking into consideration horizontal gene transfer and the differences in frequency between gene gains and gene losses. Their method computes the scenario with the smallest number of events, taking into consideration the difference in frequency between ortholog gains and losses.

**Figure 6:**
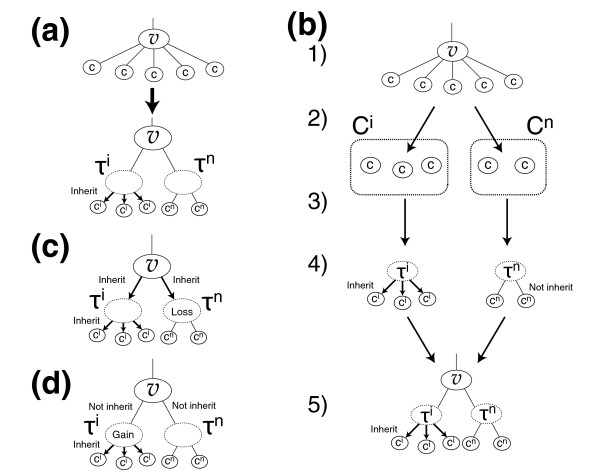
Estimation of the most parsimonious scenario of evolution in a multifurcated tree. **(a) **At internal nodes having more than two children, we insert two tentative nodes, *τ*^*i *^and *τ*^*n*^. We assume that children of *τ*^*i *^(*τ*^*n*^) always (do not) inherit the target gene from *τ*^*i *^(*τ*^*n*^). **(b) **Description of our modification for multifurcated branching (see Materials and methods). **(c) **It is assumed that the gene is lost in *τ*^*n *^when the gene is inherited from *v*. **(d) **It is assumed that the gene is gained in *τ*^*i *^when the gene is not inherited from *v*.

In this work we modified Mirkin's algorithm for multifurcated phylogenetic trees (Figure 6). At internal nodes having more than two children, we assumed that the order of child divergence with the smallest number of events was correct. Then, we insert two tentative nodes τ^*i *^and τ^*n *^as children of *v *(Figure 6a). The procedure is as follows (see Figure 6b).

In step 1, for each child node *c*, compute the number of events (gains and losses) for the case when the node inherits a gene (*e*_*i*_(*c*)) and the case when the node does not inherit a gene (*e*_*n*_(c)) using the method proposed by Mirkin and coworkers [13]. In step 2, divide the children into two groups *C*^*i *^and *C*^*n*^, based on comparison of *e*_*i*_(*c*) and *e*_*n*_(*c*) according to the following condition:

$${\{}_{C^{n}=\{c|e_{n}(c)<e_{i}(c)\}}^{C^{i}=\{c|e_{i}(c)<e_{n}(c)\}}$$

In step 3, graft the child *c *in *C*^*i *^to τ^*i *^and graft the child *c *in *C*^*n *^to τ^*n*^. In step 4, consider two cases - τ^*i *^and τ^*n *^inherit the gene (Figure 6c) and τ^*i *^and τ^*n *^do not inherit the gene (Figure 6d) - and count the events for τ^*i *^and τ^*n*^. In step 5, apply the method of Mirkin and coworkers [13] to bifurcated branching at *v *with children τ^*i *^and τ^*n*^.

If the tentative nodes τ^*i *^and τ^*n *^inherit a gene from internal node *v*, then the smallest number of events is satisfied when the gene is lost in τ^*n*^; this is because the numbers of events for children *c*^*i *^become smaller when the gene is inherited from their parent τ^*i*^, and those for children *c*^*n *^become smaller when the gene is lost in their parent τ^*n *^and not inherited from τ^*n *^(Figure 6c). If τ^*i *^and τ^*n *^do not inherit the gene from *v*, then the smallest number of events is satisfied when the gene is gained in τ^*i *^(Figure 6d). Any phylogenetic relationships within nodes in *C*^*i *^or within *C*^*n *^do not affect the smallest number of events because no event should occur among them.

### Domains inherited from the commonote

Domains existing in eukaryotes include domains inherited from the commonote, which is the common ancestor of eukaryotes, eubacteria, and archaea. Horizontal gene transfer often occurred from eukaryotes to prokaryotes, and hence it may not necessarily be true that a domain emerged in the commonotes, even if the domain is contained in both eukaryotes and prokaryotes. So we estimated the most parsimonious scenario of domain gains and losses in prokaryotes with the method described above, to find domains inherited from the commonote. As a result, domains in eukaryotes that existed in the common ancestor of eubacteria or the common ancestor of archaea were estimated, and we assume that these domains were inherited from the commonote to eukaryotes.

### Specific domains for each clade of eukaryotes

Horizontal gene transfer between major clades of eukaryotes can be disregarded [14,15,20]. Thus, the most parsimonious scenario is that a domain emerged in the last common ancestor of the existing species having proteins with the domain and only gene loss followed. We defined that the domain be specific for the clade rooted at the common ancestor.

### Generation of domain combinations

Identical domain combinations may have been independently generated in multiple clades. Thus, we estimate the parsimonious scenario with the method in the previous section by using the consensus tree of eukaryotes. Then, as in the case of specific domains, we defined a combination as being specific for the clade rooted at the common ancestor in which the combination was generated.

### Gain penalty

Frequencies of gene gains and losses are not the same, and we assume that gene losses occurred more frequently than gene gains. It is crucial for parsimonious estimation to assess the ratio of the frequency of losses to gains, and this ratio is referred to as 'gain penalty' in the method proposed by Mirkin and coworkers [13]. We implemented the gain penalty in the same way. The ratio is not the same for individual genes and domains, and hence it is difficult to estimate these values, but we found that this was not essential for the present work because all tendencies were found to be conserved when we tested values between 1 and 3. Here, we show the results when the gain penalty was set to 3 for all domains and combinations.

### Fitting to the power law distribution

To reduce the effect of noise in the data, we calculated the cumulative distribution of the degrees in each domain combination network. The cumulative distribution of the power law distribution also follows a power law, but with a different exponent. When the exponent of the original distribution is γ, the exponent for the cumulative distribution becomes γ - 1 [59]. Thus, we obtained γ by least squares fitting of the cumulative distribution.

### Estimation of specific subfamilies

Domain subfamily emergence was defined according to the species included in the subtree of the dendrogram obtained from hierarchical clustering of the domain sequences. To construct multiple alignments of each domain, we extracted sequences corresponding to the domain defined by a hidden Markov model profile in Pfam and aligned them to the profile by using HMMalign in the HMMER package. After eliminating insertions not aligned to the profiles, we carried out hierarchical clustering of the domain sequences with UPGMA using QuickTree [60], which computes a distance matrix with the method used in CLUSTAL W [61].

**Figure 7:**
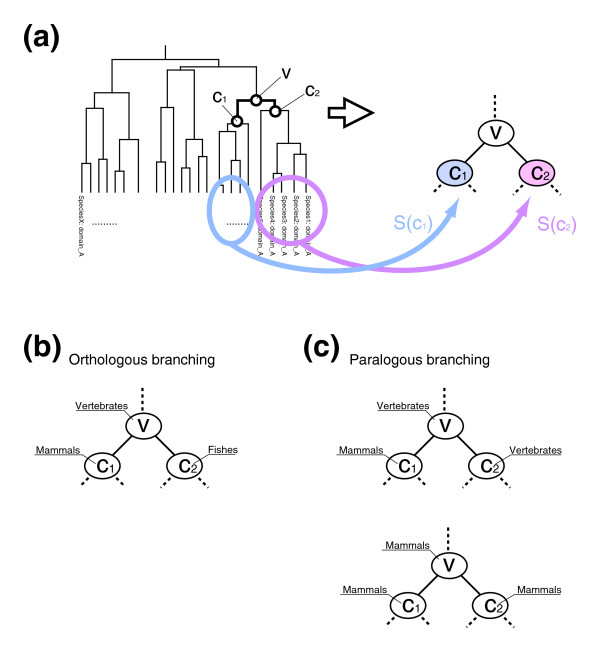
Alternative definition of domains. **(a) **Dendrogram of domains. *S*(*x*) was defined as a set of species whose domains are included in the leaves rooted at *x*. **(b) **Example of orthologous branching where *S*(*c*_1_) is a set of mammals and *S*(*c*_2_) is a set of fishes. The divergence at *v *can be correlated with the divergence of mammals and fishes. **(c) **Examples of paralogous branching. In the upper case, where *S*(*c*_1_) is a set of mammals and *S*(*c*_2_) is a set of vertebrates, it can be considered that domains are duplicated in mammals but not in other vertebrates. We ignored serial duplication such as in the bottom case.

Domains can be classified by hierarchical clustering based on sequence similarity. However, it is impossible to define a general threshold of sequence similarity to divide subfamilies for various domains. Thus, taking into account the generally accepted assumption that subfamilies were created by duplication of paralogs, we comprehensively and automatically defined subfamilies of Pfam domains by considering paralogous duplications of the domains based on the hierarchical clustering of domain sequences. Comparing the phylogenetic tree of eukaryotes *T*^*Species *^and the dendrogram *T*^*Domain *^obtained by hierarchical clustering, we systematically defined the emergence of subfamilies of the respective domains. Each leaf *d *of the tree *T*^*Domain *^represents a domain sequence of a species *s*_*d*_. Let *S*(*x*) be a set of such species for all leaves of a subtree *T*_*x *_^*Domain *^rooted at *x *as follows (also see Figure 7a):

$$S(x):=\{s_{d}|d\in leaves(T_{x}^{Domain})\}.$$

Then, a branch at an internal node *v *on the dendrogram can be one of the following two types, namely orthologous branching by the divergence of species

*S*(*c*_1_) ∩ *S*(*c*_2_) = ∅,

and paralogous branching by gene duplication

*S*(*c*_1_) ∩ *S*(*c*_2_) ≠ ∅,

where *c*_1 _and *c*_2 _are the children of *v *(Figure 7b,c). Here, we defined subfamilies as having diverged with gene duplication, and we only considered the first duplication if serial duplications occurred more than once in the same ancestral species. Therefore, we extracted the internal nodes *v *at paralogous branches satisfying the following condition:

lca(*S*(*c*_2_), *T*^*Species*^) ∈ ancestors(*c*_1_, *T*^*Species*^)

Where *lca*(*S*, *T*^*Species*^) denotes the last common ancestor of a set of species *S*, and ancestors(*s*, *T*^*Species*^) denotes the set of all nodes in the path from the root to the parent of node *s *in the phylogenetic tree *T*^*Species *^(all ancestral species at each branch of the clade to species *s *in evolution). Then, the time when the subfamily diverged was estimated to be *lca*(*S*(*c*_1_), *T*^*Species*^). Because the domain sequences were hierarchically classified, subfamilies were defined hierarchically.

## Additional data files

The following additional data are available with the online version of this paper. Additional data file 1 contains a figure showing detailed phylogenetic relationship among 47 eukaryotes. Additional data file 2 contains a figure showing the number of combination partners of group-specific domains in deuterostomes. Additional data file 3 contains a figure showing the number of combination partners of group-specific domains in invertebrates. Additional data file 4 contains a figure showing the number of combination partners of group-specific domains in fungi. Additional data file 5 contains a figure showing the number of combination partners of group-specific domains in protists. Additional data file 6 contains a figure showing the number of combination partners of group-specific domains in plants. Additional data file 7 contains tables showing the statistics of domain assignments for eukaryotes (Supplementary Table 1) and all results of history reconstruction (Supplementary Table 2).

## Supplementary Material

Additional data file 1 Provided is a figure showing detailed phylogenetic relationship among 47 eukaryotes. Click here for file

Additional data file 2 This figure illustrates the difference in the number of combination partners among each group-specific domain in extant deuterostomes. Click here for file

Additional data file 3 This figure illustrates the difference in the number of combination partners among each group-specific domain in extant invertebrates. Click here for file

Additional data file 4 This figure illustrates the difference in the number of combination partners among each group-specific domain in extant fungi. Click here for file

Additional data file 5 This figure illustrates the difference in the number of combination partners among each group-specific domain in extant protists. Click here for file

Additional data file 6 This figure illustrates the difference in the number of combination partners among each group-specific domain in extant plants. Click here for file

Additional data file 7 Provided are tables showing the statistics of domain assignments for eukaryotes (Supplementary Table 1) and all results of history reconstruction (Supplementary Table 2). Click here for file
