# Supplementary figures and images for: Evolutionary history and functional implications of protein domains and their combinations in eukaryotes
Source: Genome Biol. 2007 Jun 25;8(6):R121. doi: 10.1186/gb-2007-8-6-r121 (PMC2394772; doi:10.1186/gb-2007-8-6-r121)

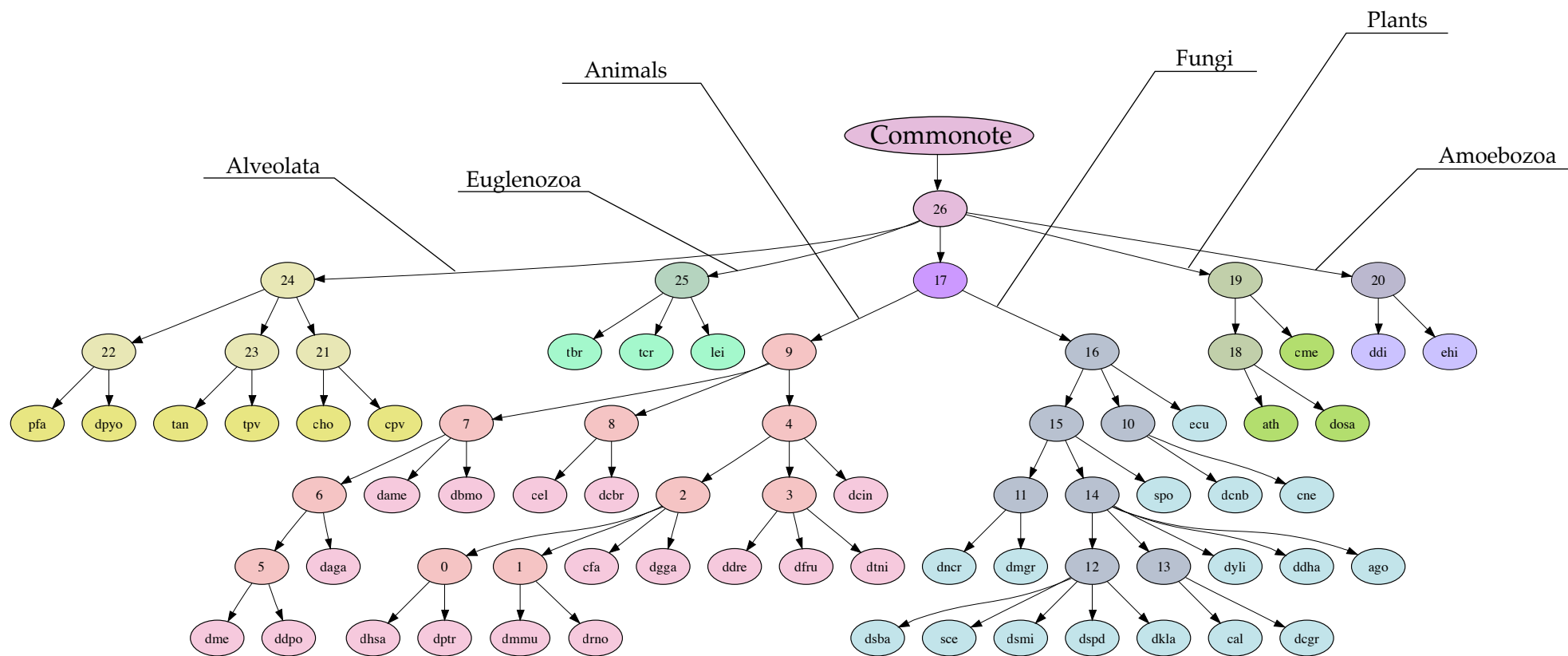

Supplement: Additional data file 1 — Provided is a figure showing detailed phylogenetic relationship among 47 eukaryotes. [file gb-2007-8-6-r121-S1.pdf]

# Deuterostomes

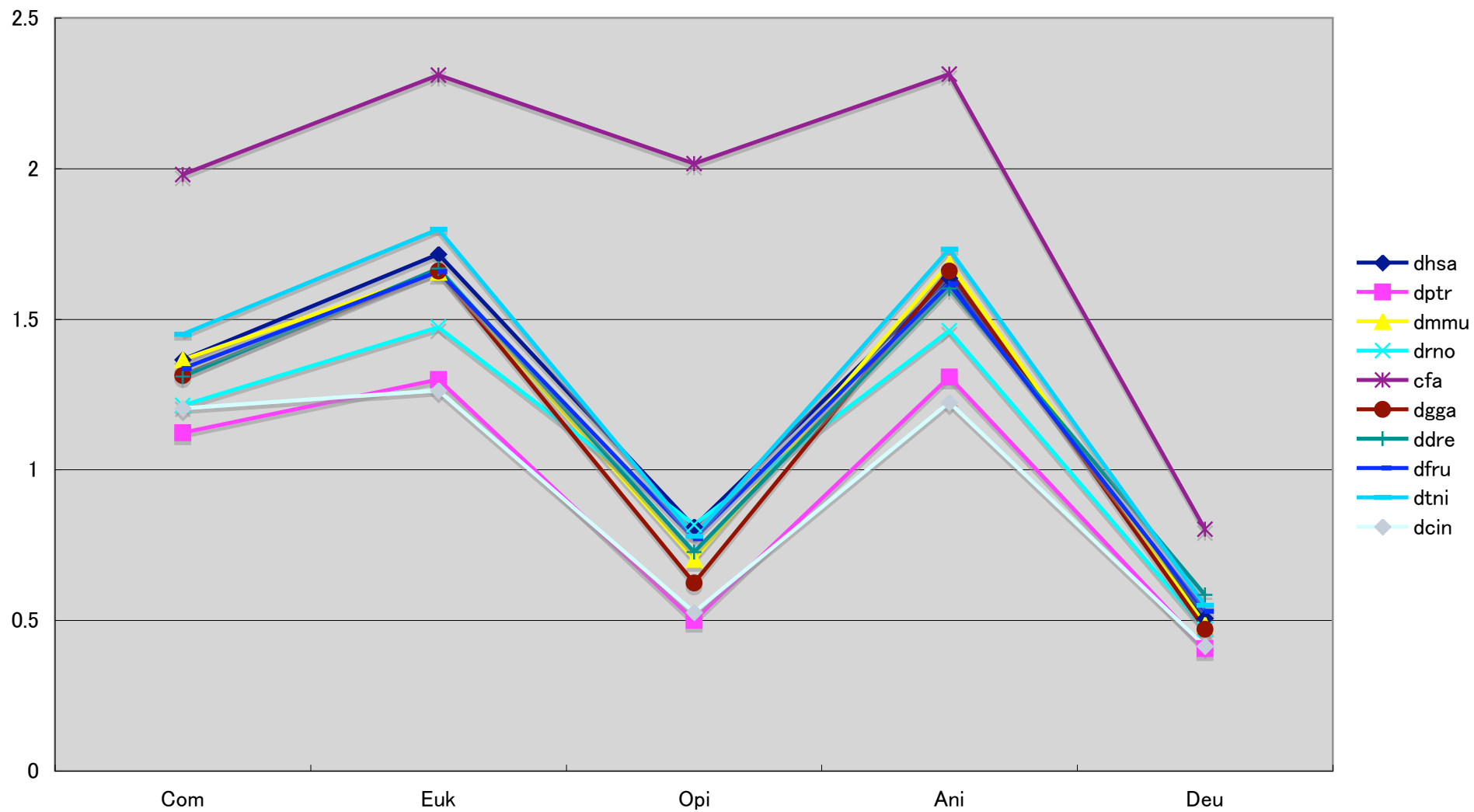

Supplement: Additional data file 2 — This figure illustrates the difference in the number of combination partners among each group-specific domain in extant deuterostomes. [file gb-2007-8-6-r121-S2.pdf]

Invertebrates (Insects + Nematoda)

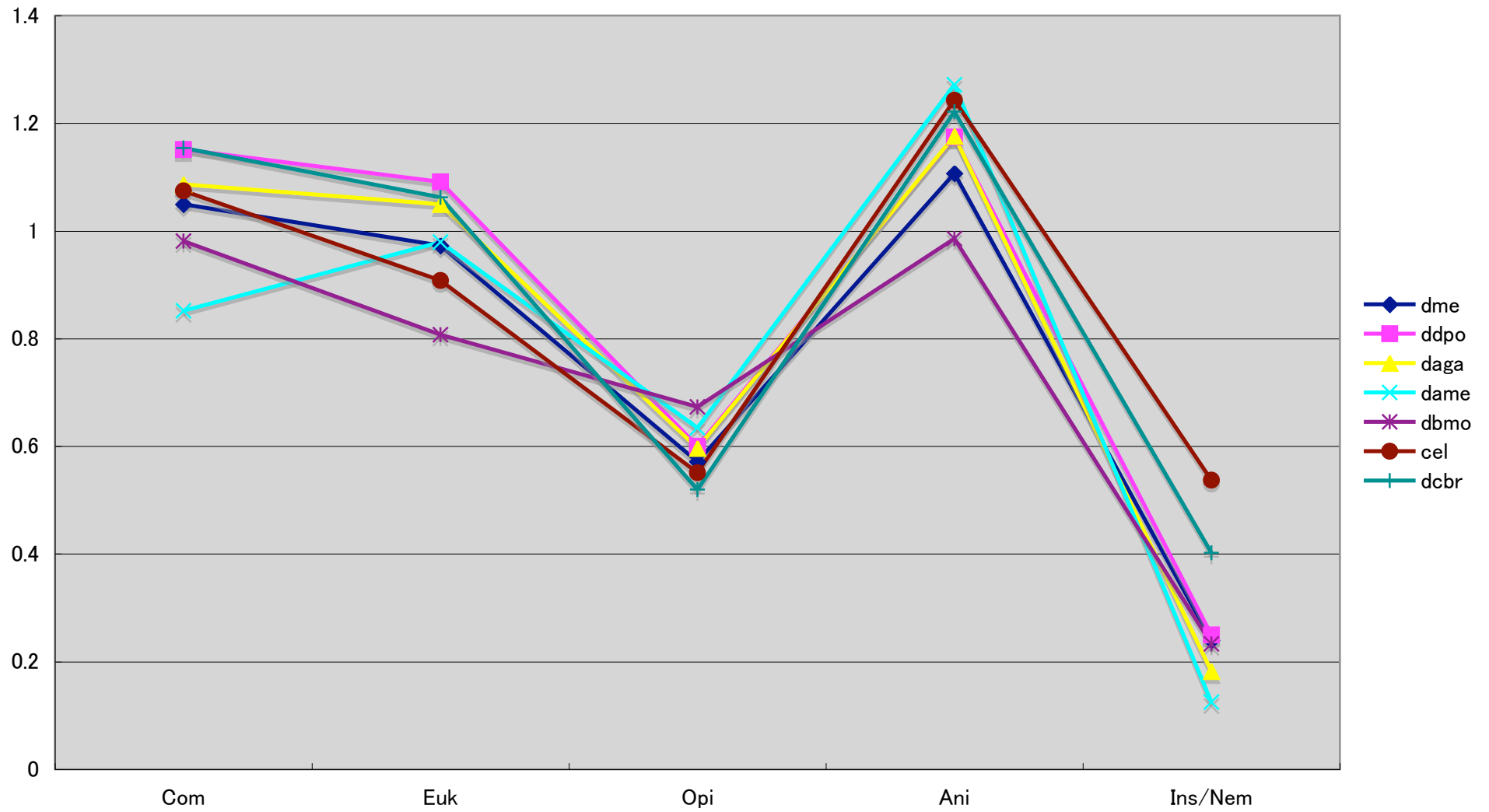

Supplement: Additional data file 3 — This figure illustrates the difference in the number of combination partners among each group-specific domain in extant invertebrates. [file gb-2007-8-6-r121-S3.pdf]

# Fungi

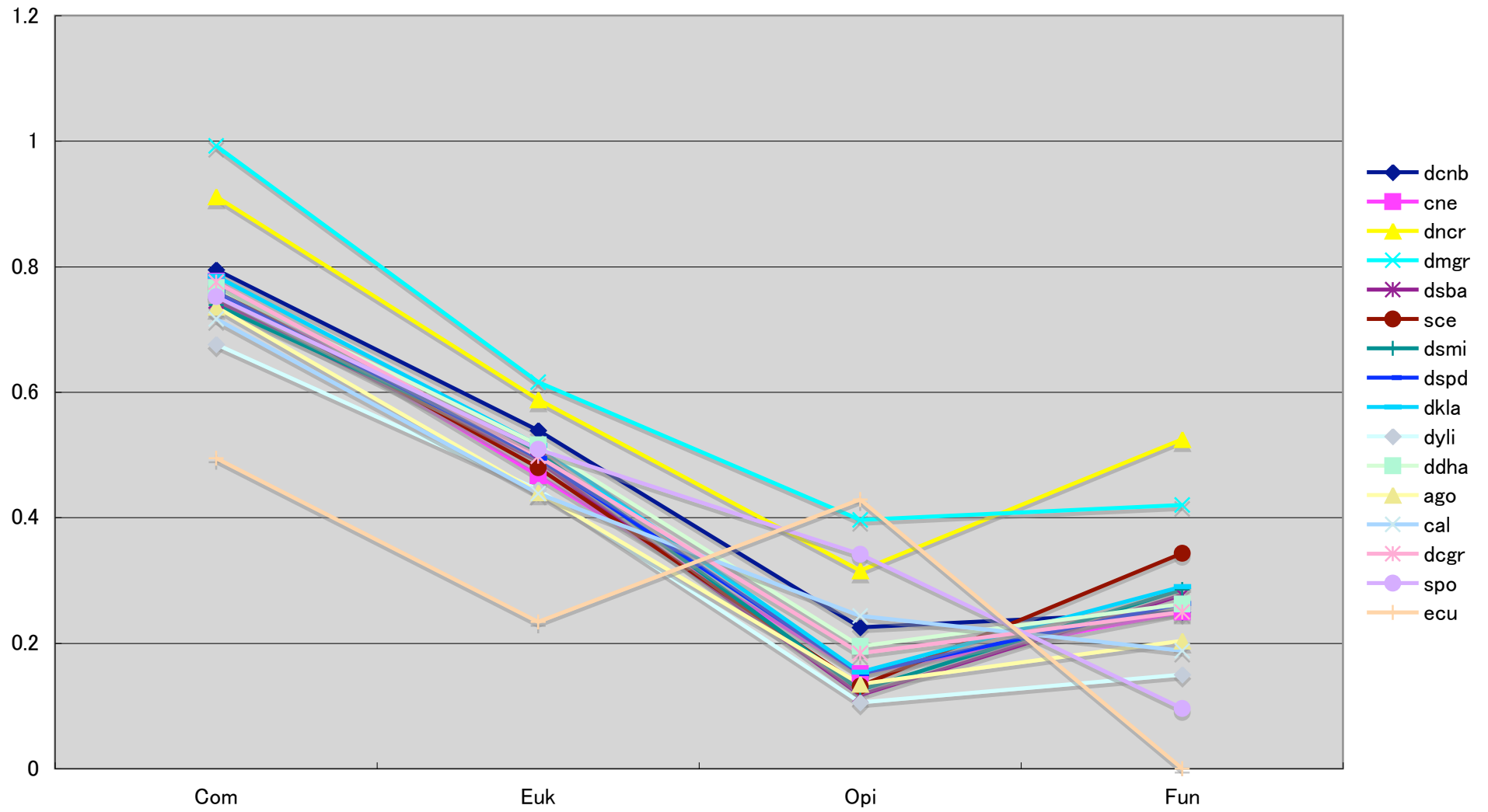

Supplement: Additional data file 4 — This figure illustrates the difference in the number of combination partners among each group-specific domain in extant fungi. [file gb-2007-8-6-r121-S4.pdf]

# Protists

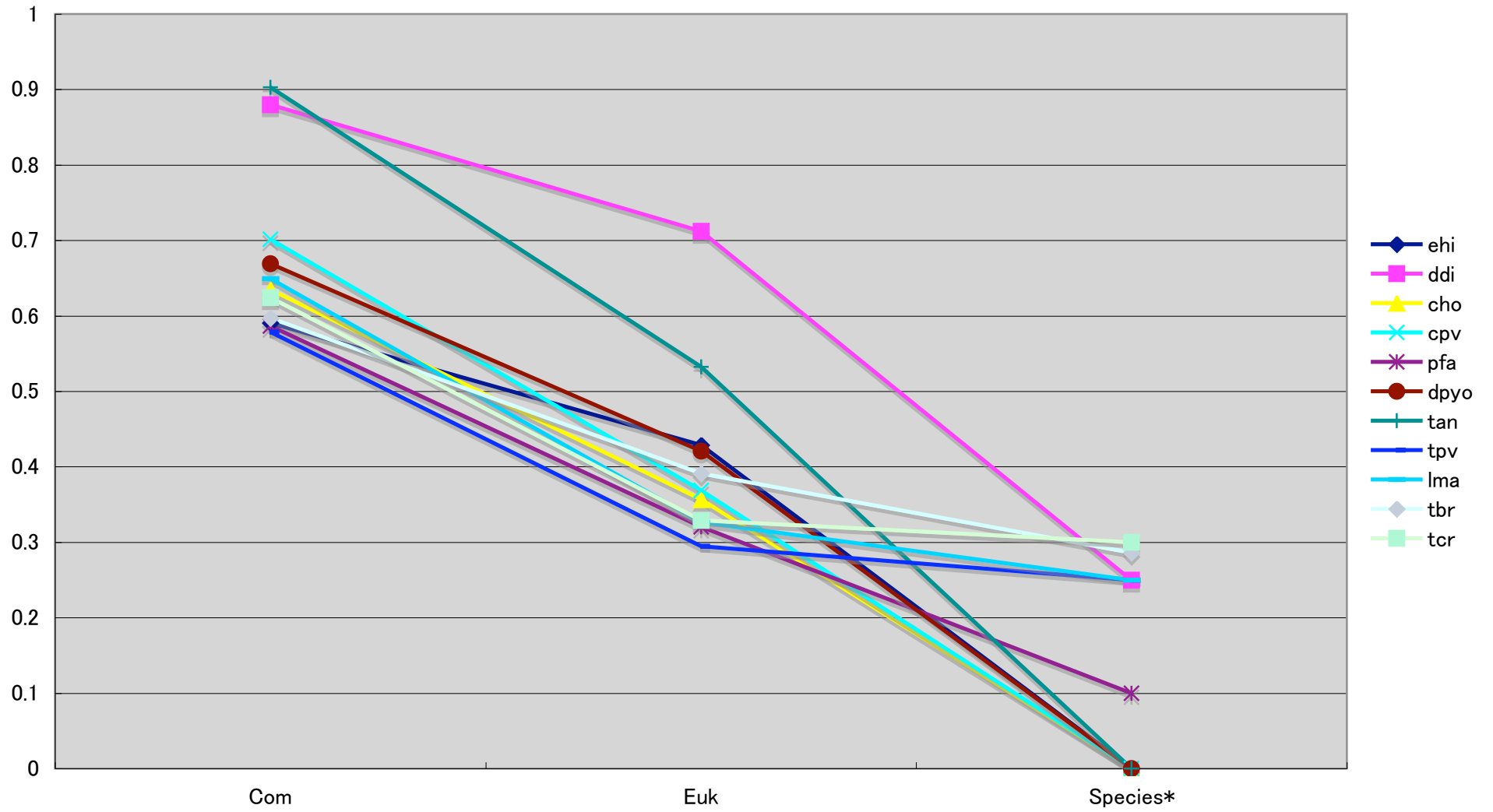

Supplement: Additional data file 5 — This figure illustrates the difference in the number of combination partners among each group-specific domain in extant protists. [file gb-2007-8-6-r121-S5.pdf]

# Plants

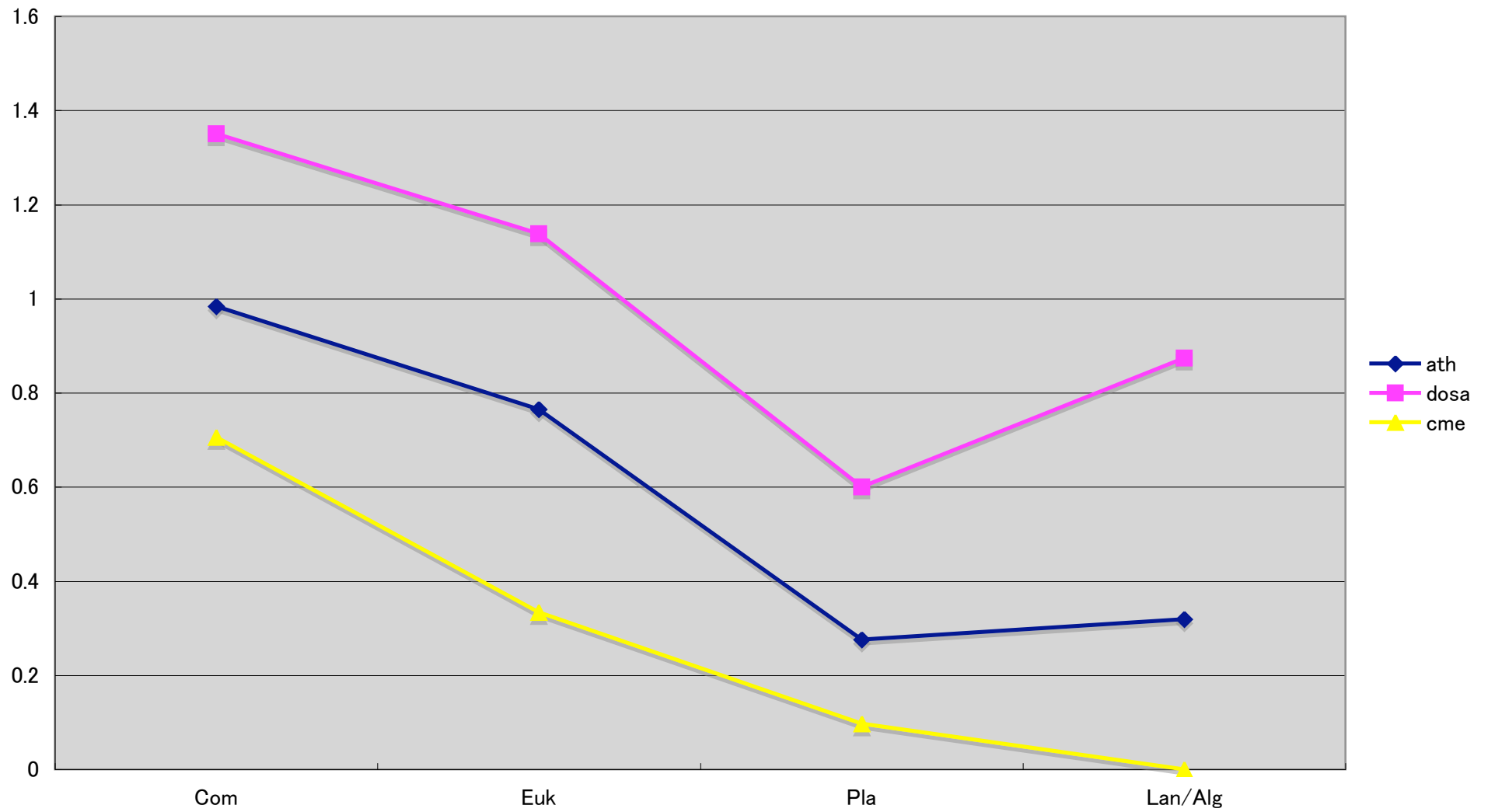

Supplement: Additional data file 6 — This figure illustrates the difference in the number of combination partners among each group-specific domain in extant plants. [file gb-2007-8-6-r121-S6.pdf]
